# The effectiveness and perception of the use of sulphadoxine-pyrimethamine in intermittent preventive treatment of malaria in pregnancy programme in Offinso district of ashanti region, Ghana

**DOI:** 10.1186/1475-2875-10-385

**Published:** 2011-12-29

**Authors:** Emmanuel Osei Tutu, Bernard Lawson, Edmund Browne

**Affiliations:** 1 Department of Theoretical and Applied Biology, Faculty of Biosciences, Kwame Nkrumah University of Science and Technology, Kumasi, Ghana; 2 Department of Community Health, School of Medical Sciences, Kwame Nkrumah University of Science and Technology, Kumasi, Ghana; 3 Faculty of Public Health and Allied Sciences, Catholic University College of Ghana, Sunyani, Ghana

## Abstract

**Background:**

Malaria in pregnant women has been shown to be associated with low birth weight, stillbirth and mortality in newborns. The WHO has adopted the use of sulphadoxine-pyrimethamine (SP) to control malaria, a disease which worsens the plight of pregnant women leading to low birth weight, stillbirths and increased neonatal mortality. The present study assessed the effectiveness of SP and perception of its use in pregnant women in Offinso district (Ashanti Region), Ghana.

**Method:**

Pregnant women, gestational age 32 weeks prior to term, were studied from November 2006 to October 2007. Their haemoglobin levels (Hb), parasitaemia and other quantitative determinants were assessed. In-depth interviews (IDIs) and focus group discussions (FGDs) were used to assess the perception of SP usage and its effectiveness.

**Results:**

Of the 306 study participants, 92 (30%) took one dose, 100 (33%) two doses and 114 (37%) three doses of SP, respectively. There was significant association between gravidity and SP dosage taken (Pearson *χ*^2 ^= 18.9, *p *< 0.001). Although adverse effects were produced in 113 (i.e. 37%) of the pregnant women, no significant difference was observed with regard to the dosage of SP taken (Pearson's *χ*^2 ^= 2.3, *p *≥ 0.32). Peripheral parasitaemia was present in 47 (15%) of the subjects. There was a poor negative relationship of doses of SP with parasitaemia (*r *= -0.07, *p *≥ 0.24). Mean Hb was 11.3 ± 1.6 g/dl, with 118 (39%) of the subjects anaemic (Hb < 11.0 g/dl), whilst 187 (61%) were normal (Hb ≥11.0 g/dl). Significant positive correlation of SP use with Hb level (*r *= 0.15, *p *< 0.008) was observed. SP use reduced malaria and anaemia prevalence, contributed to reduced maternal morbidity with mild side effects being reported.

**Conclusions:**

This study points to the effectiveness of IPTp using SP as an evidence-based measure for control of malaria and malaria-related anaemia in pregnancy. Therefore, the Ghana Health Service should improve current programme strategies to increase the proportion of pregnant women who take three doses of SP, paying attention to improved face-to-face health education, focussed antenatal care and better social mobilization.

## Background

In malaria endemic communities, pregnant women and children are more vulnerable to malaria infection. Its effects in pregnancy include chronic anaemia, acute severe anaemia, miscarriage/forced abortion and pre-term delivery in the mother, and in the foetus and newborn; low birth weight, congenital malaria, stillbirth, perinatal and neonatal deaths [1,2]. In sub-Saharan Africa, the major detrimental effect of malaria infection is low birth weight (LBW) and maternal anaemia [1,3,4]. Malaria infection during pregnancy has been estimated to cause 75,000-200,000 infant deaths each year in stable transmission areas [3,5].

To control malaria in pregnancy, the intervention by the WHO with the use of sulphadoxine-pyrimethamine (SP) in intermittent preventive treatment of malaria programme (IPTp) in pregnancy has been established and documented in improving birth weight of neonates and reducing maternal morbidity [1,6-8]. A recent study by Tutu et al. [9] elucidated on the effect of SP on pregnant women and confirmed other studies [4,10-13] that SP reduces malaria-related anaemia in pregnant women as compared to those who did not take SP during pregnancy. However, the problem of maternal morbidity and neonatal low birth weights is still persistent in the country. The present study, therefore, updated information on the use of SP in IPTp in pregnant women and assessed the knowledge and the perceptions of its use in Offinso District, Ghana.

## Method

### Study area

**Figure 1:**
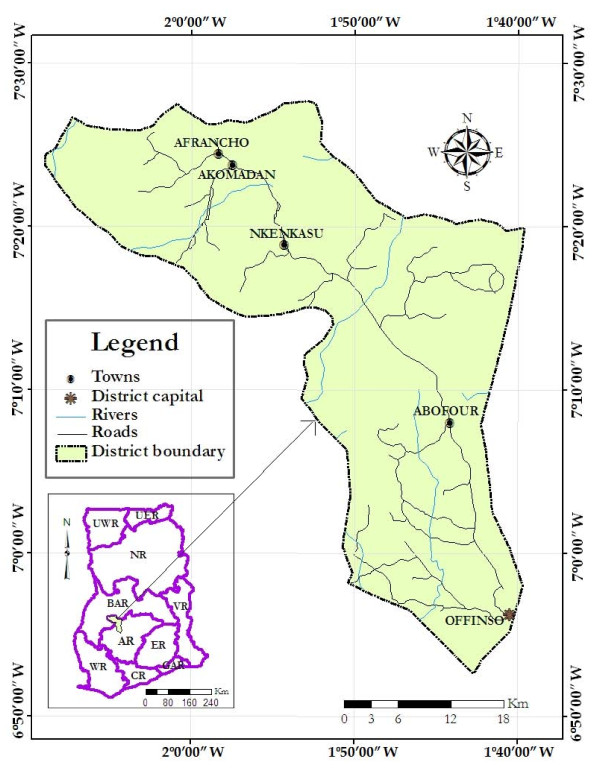
**Map of Offinso District showing the study towns (*Source: Prepared using ArcGIS software, 2008*)**.

The study area has been described elsewhere [9], but briefly, the study was conducted in Offinso District, one of the 27 administrative districts in the Ashanti Region of Ghana (Figure 1). The study was carried out in six health facilities that provide antenatal, delivery and postnatal services in the district, and communities where these health centres are situated. The health facilities were St. Patrick's Hospital (Offinso), District Assembly Maternal and Child Health Care Centre (Offinso), Nkenkaasu Hospital, Abofour Health Centre, Akomadan Health Centre and A.M.E. Zion Health Centre (Afrancho).

### Study design and population

The study was an analytical type with a cross-sectional design. Pregnant women attending antenatal clinic in the six health facilities and who were 32 weeks of gestation to term and had taken SP were included in the study. Pregnant women with gestational age < 32 weeks irrespective of their IPTp status were excluded from the study.

### Sample size

A sample size of 296 was estimated for the cross sectional study. The prevalence of anaemia in pregnant women was 50% [14] and in reducing maternal anaemia by 5% through the use of SP at the power of 80% and 5% significance, a sample size of 296 was required for the cross sectional study (using Statcalc, Epi info software 2002, version 6). However, a little over the sample size, 306 data was collected.

### Sampling method

Stratified sampling method was used in selecting the health facilities while convenience (purposeful) sampling method was used to sample the respondents. The total number of pregnant women seen yearly in each health facility was made as a fraction of the total number of pregnant women seen in all these health facilities annually. The proportion for each health facility was used to determine the number of pregnant women to be sampled from that health facility.

### Laboratory investigations and qualitative study

The finger-prick method was used to collect blood for determination of haemoglobin (Hb) levels and parasitaemia as previously discussed [9]. Focus Group Discussions (FGDs) and in-depth interviews (IDIs) were conducted in the communities. This qualitative study was undertaken to assess the knowledge and understanding of study participants on SP in IPTp programme and possible outcomes of treatment. Three assemblymen, three chiefs, one queen mother, an opinion leader as well as nine chemical sellers in the district were interviewed. Four focus group discussions (FGDs) of "horse shoe" type were conducted with pregnant women. Two were conducted in Offinso Central communities and the other two in Akomadan and Afrancho communities. The pregnant women were assigned to one or the other of two categories namely: pregnant women who were less than twenty years of age and those who were twenty years or older.

### Ethical consideration

Information on the study was provided to the District Health Administration, the District Assembly and the opinion leaders in the study communities. Permission to undertake the study was obtained from these stakeholders. Ethical clearance was sought and obtained from the Ghana Health Service and the School of Medical Sciences (SMS) of the KNUST Ethics Committees. Each study participant, after being briefed and offered the opportunity to ask questions about the study, was provided with individual informed written consent form to sign or thumbprint. The written consent forms and participant information forms were kept separately from the data collection tools.

### Definitions and groupings

IPTp was defined as administration of a curative anti-malarial treatment dose of SP at predefined intervals (between 16-36 weeks) to asymptomatic pregnant women during antenatal clinic (ANC), who are at risk of malaria, regardless of whether or not they are parasitaemic at the time of visit. Haemoglobin levels (Hb < 11 g/dl) and (Hb < 7 g/dl) in pregnant women were considered as moderate anaemia and severe anaemia, respectively. Parasitaemia was defined as the presence of asexual stage parasites in thick smears. Gravidity was categorized into primigravidae (women in their first pregnancies), secundigravidae (women in their second pregnancies) and multigravidae (women in their third, fourth or more pregnancies) as discussed in elsewhere in Tutu et al. [9].

### Statistical analysis

The study participants were given an identification number, identifying village, house number and a randomly computer generated digit. All answers were numerically coded on the questionnaire and laboratory results entered into Microsoft Office Access, 2003 version. Data were analysed using Stata version 10.0 (Stata Corporation 4905, Lakeway Drive College Station, Texas 77845, USA). Averages and 95% confidence interval (CI) were used for summarizing of results. Frequencies and percentages were used to compare number of participants associated with the use of SP, parasitaemia, haemoglobin levels etc. Analyses of differences in proportions were done using Pearson's chi-square test or Fisher's exact test where appropriate. Analysis of variance (ANOVA) with Bonferroni measure of comparison was used to measure the differences in means and Pearson correlation coefficient used to determine the relations between variables. For all statistical tests in this IPTp study, p < 0.05 was considered significant.

Responses from the qualitative survey were tape-recorded and transcribed. The data were manually analysed along defined themes including knowledge of SP in IPTp and its effects and benefits in preventive treatment of malaria in pregnancy.

## Results

### Baseline characteristics of the study population

**Figure 2:**
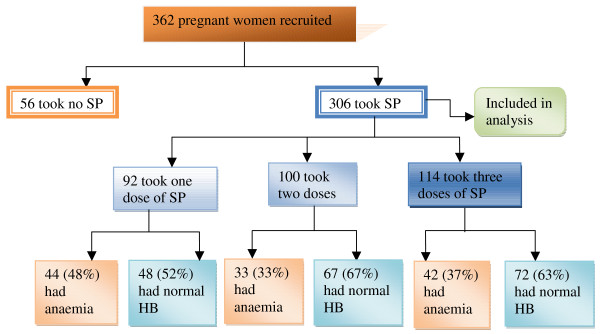
**Trial profile of pregnant women recruited for the study**.

The study recruited 362 pregnant womenof which 56 of them took no SP and were excluded from the analysis (Figure 2). The mean age of the 306 study women who were included in the analysis and took SP (Figure 2) was 27 ± 6 years with a 15 year old being the youngest and a 46 year old, the oldest. Over 50% of the women were between 20-29 years with less than 10% below 20 years (Table 1). Primigravidae constituted 18% while secundigravidae and multigravidae made up 24.5% and 57.5% respectively of the study participants. Traders (35.3%) formed majority of the pregnant women studied. Artisans including dressmakers, hair dressers, potters and tie-dyers, made up 16% of the study women. Civil servants including teachers, nurses, secretaries, etc. made up 3.9% while the unemployed made up 19% of study women (Table 2). Property highly owned by the pregnant women was radio (85.3%), while motorbikes were least (10.1%) owned by them. Possession of mosquito nets among the pregnant women was 59.8% with 56.5% being insecticide-treated nets (ITNs). However, less than 50% of the women slept in ITNs (Table 2).

**Table 1:** Background Characteristics of pregnant women

Age	N (306) (%)
≤19	27 (8.82)

20-29	173 (56.54)

30-39	102 (33.33)

≥40	4 (1.31)

**Gravida**	

Primigravidae	55 (18.0)

Secundigravidae	75 (24.5)

Multigravidae	176 (57.5)

**Marital Status**	

Married	149 (48.7)

Single	157 (51.3)

**Educational Level**	

None	76 (24.8)

Primary	67 (21.9)

Middle/J.H.S.	133 (43.5)

Secondary	23 (7.5)

Tertiary	7 (2.3)

**Religion**	

Christian	218 (71.24)

Moslem	75 (24.51)

Traditionalist	13 (4.25)

N = total number of study subjects

**Table 2:** Socioeconomic status of the pregnant women

Occupation	N (306) (%)
Farmer/housewife	79 (25.8)

Trader	108 (35.3)

Artisan	49 (16.0)

Civil servants	12 (3.9)

Unemployed	58 (19.0)

**Own Properties**	

Car	53 (17.3)

Motorbikes	31 (10.1)

Bicycles	155 (50.7)

Radio	261 (85.3)

Television	154 (50.3)

Sleep on bed	264 (86.3)

Windows netted	171 (55.9)

Have mosquito nets	183 (59.8)

Have ITNS	173 (56.5)

Sleep in ITNS	145 (47.4)

Latrine at home	118 (38.6)

**Source of drinking water**	

Pipe borne water	159 (52.0)

Well water	78 (25.5)

Bore-hole water	100 (32.7)

River water	43 (14.0)

**Animals kept at home**	

Goats	54 (17.6)

Sheep	55 (18.0)

Poultry	188 (61.4)

Housing type	

Blocks with Al roofing	86 (28.1)

Mud/Bricks with thatches/Al roofing	220 (71.9)

The average number of persons per room was 3 ± 1.5 with the least occupant being one and the highest number of occupants being 13. Twenty eight percent (86 women) lived in houses built with cement blocks and roofed with aluminium sheets, whilst 72% (220) of them lived in mud and brick houses with either aluminium roofing sheets or thatches as roof cover (Table 2). Seventy four (24%) used traditional medicine which included preparation of herbs and the bark of trees for enemata and those added to foods eaten, which they reported to be helpful as diuretics, for control of nausea and prevention of constipation. However, these herbs had no anti-malarial effects.

### Use of SP and adverse effects

Nearly a third, 92 (30%) took one dose, 100 (33%) two doses and 114 (37%) three doses of SP, respectively (Figure 2). Multigravid women (57.5%) took more doses of SP as compared to 24.5% and 18% of secundigravid and primigravid women respectively (Table 3). A little over a third, 113 (37%) of the pregnant women reported adverse reactions (including nausea, general malaise, body weakness, vomiting etc.) with the intake of SP; however, these effects showed no significant association with the number of doses of SP taken (Pearson's *χ*^2 ^= 2.3, *p *≥ 0.32).

**Table 3:** Doses of SP taken by pregnant women

*Gravida*	*Dose 1*	*Dose 2*	*Dose 3*	*Total*
Primigravida	12 (13.04%)	27 (27.0%)	16 (14.0%)	**55 (18.0%)**

Secundigravida	13 (14.13%)	27 (27.0%)	35 (30.7%)	**75 (24.5%)**

Multigravida	67 (72.83%)	46 (46.0%)	63 (55.3%)	**176 (57.5%)**

**Total (100%)**	**92 (100.0%)**	**100(100.0%)**	**114(100.0%)**	**306(100.0%)**

### Haemoglobin level and SP in pregnant women

The mean haemoglobin (Hb) level was 11.3 ± 1.6 g/dl (95% CI: 11.1 to 11.4). One hundred and eighteen women (39%) had moderate anaemia (Hb < 11.0 g/dl) whilst most of the pregnant women 187 (61%) had normal haemoglobin level (Hb ≥ 11.0 g/dl). There was no significant association between haemoglobin level and gravida of pregnant women (Pearson's *χ*^2 ^= 4, *p *≥ 0.14). Comparing Hb level with other socioeconomic parameters including education, occupation, religion, marital status and use of ITN did not show any significant associations (Pearson's *χ*^2 ^= 0.7, *p *≥ 0.9; Pearson's *χ*^2 ^= 1.8, *p *≥ 0.6; Pearson's *χ*^2 ^= 6.1, *p *≥ 0.1; Pearson's *χ*^2 ^= 1.3, *p *≥ 0.3; Pearson's *χ*^2 ^= 1.2, *p *≥ 0.3 respectively). There was no significant association between parasitaemia and haemoglobin level of the pregnant women (Fisher's exact = 0.87).

Haemoglobin level and the doses of SP taken, however, did show significant association (Pearson's *χ*^2 ^= 182.2, *p *< 0.02). Haemoglobin levels among those who took various doses of SP did show significant difference (*p *< 0.007). By Bonferroni method of comparison, the haemoglobin level of the pregnant women who took the first dose of SP was, on average, 0.65 (*p *< 0.017) and 0.62 (*p *< 0.019) significantly lower than those who took the second and third doses of SP, respectively. The haemoglobin level of those who took the third dose of SP was, on average, (0.03) lower than those who took the second dose of SP but the difference was not significant (*p *≥ 1). However, there was significant positive correlation of SP with haemoglobin level (*r *= 0.15, *p *< 0.008).

### Parasitaemia and SP in pregnant women

Forty seven (15%) of the pregnant women studied had parasitaemia. However, there was a weak association between gravida and parasite density (Pearson's *χ*^2 ^= 10.8, *p *< 0.09). Multigravid women (26/47) were highly parasitaemic as compared to primigravid (10/47) and secundigravid women (11/47); nevertheless, parasite density of ≥ 5000 per μl of blood was more found in primigravid women as compared to the secundigravid women, but the multigravid women recorded none (Table 4).

**Table 4:** Parasite density (per μl of blood) of pregnant women

*Gravida*	*None*	*Parasite density (per μl of blood)*			*Total*
				
		*1-1999*	*2000-4999*	*> = 5000*	
Primigravida	45 (17.4%)	6 (15.0%)	1(33.3%)	3 (75.0%)	**55 (18.0%)**

Secundigravida	64 (24.7%)	9 (22.5%)	1(33.3%)	1 (25.0%)	**75 (24.5%)**

Multigravida	150 (57.9%)	25 (62.5%)	1 (33.3%)	0 (00.0%)	**176 (57.5%)**

**Total (100%)**	**259 (100.0%)**	**40 (100.0%)**	**3 (100.0%)**	**4 (100.0%)**	**306 (100.0%)**

Pearson's correlation showed negative relations of the parasitaemia level with the number of doses of SP taken but these were not significant (*r *= -0.07, *p *≥0.24) using Bonferroni method of comparison.

### Knowledge of SP in IPTp and its effects

IDIs conducted with opinion leaders and chemical sellers to obtain information concerning their knowledge on IPTp showed that many of the chemical sellers did not have much knowledge on SP-IPTp although they knew what SP was. FGDs with pregnant women also revealed that few of them really knew which drug they were taking and why they took it.

*"I have heard about that one and some of them even come here to buy it because when it was given to them in the hospital they realized it was good"... we know it as Fansidar and in the hospital it's Malafan"*. [Chemical seller, Aboasu]

*"Yes. I heard that they are made to take it right there for 3 times. So if a pregnant woman does not attend the antenatal clinic and faces any complications then it is her own problem"*. [Chemical seller, Afrancho]

*"A doctor from Offinso came to talk to us about it and I think they take it for 3 times before delivery and it is helping them because I have not heard of premature birth since it started"*. [Queen mother of Afrancho]

When a chemical seller was asked whether he had heard about SP, he said he had not heard much about it. "*I heard that the pregnant women go to the hospital for some chloroquine tablets*". [Chemical seller, Offinso] This also shows that some people are still ignorant about what is happening in relation to using SP in IPTp.

A pregnant woman added: *"When you are four months old and you feel the baby quickening, she, the nurse, gives you a drug that will protect both the mother and the baby from malaria. And you are to take the drug every month for three times*". [FGD, pregnant women]

Most of the chemical sellers knew about Fansidar^® ^and Malafan^® ^but did not know much about SP use in IPTp. Some also did not know that Fansidar^® ^is the same as SP. The information gathered indicated that the pregnant women did not observe any major adverse effects associated with intake of SP. The adverse effects mentioned, however, and which were considered minor were vomiting, nausea and body weakness with isolated cases of itching and palpitation. *"When I took the drug, I vomited and became very weak"*. [FGD, pregnant women]

Some of the pregnant women said they experienced minor problems such as nausea, vomiting, body weakness etc. when they took it for the first time but with the subsequent doses they did not experience any adverse effects. *"When I took the first dose, I vomited but with the second dose I did not. She, (the midwife) told me to continue taking it. And before I took the drug someone had already explained to me that with the first dose you can vomit or become weak. So I was aware before coming in for the drug"*. [FGD, pregnant women]

Another pregnant woman added: *"When I took the first dose, I was bodily weak, nauseous and even vomited later on, but when I took the second dose nothing happened"*. [FGD, pregnant women] *"I didn't vomit when I took the first dose but with the second dose and the third dose, I vomited"*. [FGD, pregnant women] *"When I took the first dose, I felt weak and lost appetite"*. [FGD, pregnant women] *"I felt weak and this continued when I took the second dose but I told the midwife about it and she encouraged me to take the third dose"*. [FGD, pregnant women]

### Benefits of IPTp

The IDIs and FGDs revealed that the IPTp programme was effective. Most of the respondents admitted that the rate at which the pregnant women reported sick had reduced. They also admitted that the benefits of IPTp for mothers also extends to their unborn babies, *"It protects us from getting malaria and anaemia"*. [FGD, pregnant women] "*It has some benefits because now we hardly hear complications in pregnancy and it has also reduced stillbirths*". [Chemical seller, Akomadan].

In-depth interviews indicated that IPTp with SP had benefited pregnant women and their babies since they hardly contracted malaria and the birth weights of their babies had improved. One chief said *"Yes, only if the pregnant women will follow the instructions and take it" *[Chief of Aboasu].

## Discussion

Malarial infection during pregnancy increases the risks of severe sequelae for the pregnant woman and the risk of delivering a low birth weight baby. The present study showed that the IPTp with SP programme is helpful in reducing malaria-related maternal anaemia and *P. falciparum *parasitaemia in pregnant women as discussed by various studies and reports [1,6,9,10,15,16]. Increased doses of SP were associated with increased Hb levels which do confirm the previous study done [9]. Among the gravida in the pregnant women, Hb level did not show any significant association with use of SP and thus, nearly two third, 61% of them had normal Hb level (Hb ≥11.0 g/dl) with no recording of severe anaemia (Hb < 7 g/dl). This however, contradicts the previous study [9] significant association of Hb level with gravidity since not all the study participants did take SP. Thus, the use of SP in pregnancy improves Hb levels in them.

There was reduced parasitaemia 15% (47/306) in the pregnant women, which could be attributed to the increased doses of SP taken. The SP negatively correlated with parasitaemia but was not significant (*r *= -0.07, *p *≥0.24). In the qualitative studies however, SP was commented on to protect against malaria-related anaemia in pregnancy; *"It protects us from getting malaria and anaemia"; *said one pregnant woman in the FGD *"Yes, only if the pregnant women will follow the instructions and take it" *commented a chief. Though parasitaemia was high among the multigravidae as compared to the secundigravidae and primigravidae, the primigravid women recorded higher parasite densities (≥5000 per μl of blood) as compared to secundigravid and multigravid pregnant women. This indicates the high susceptibility of the primigravid women to malaria and possible development of parasite resistance to the SP as reported in the previous study [9].

There were lots of chequered reactions on taking the doses of SP as commented by some of the pregnant women in the FGD: *"When I took the first dose, I was bodily weak, nauseous and even vomited later on, but when I took the second dose nothing happened"; "I didn't vomit when I took the first dose but with the second dose and the third dose, I vomited"*.; *"When I took the first dose, I felt weak and lost appetite"*. However, these adverse effects were not serious enough to pick up on SP in pregnancy and not significant with the number of doses of SP taken, hence, supports the mild effects of SP reported in other studies [1,9,10,15,17].

SP use as a preventive treatment drug for malaria in pregnancy was not well known among the people in the communities (particularly the chemical sellers) and, thus, they were less informed and educated about it "*I have heard about that one and some of them even come here (the drug store) to buy it because when it was given to them in the hospital they realized it is good", "... we know it as Fansidar and in the hospital it's Malafan"*.

A comment from one chemical seller: "*I heard that the pregnant women go to the hospital for some chloroquine tablets*" indicates that most of the people are less knowledgeable about the use of SP in the IPTp and not following the instructions on administering the drug could lead to abuse by pregnant women and the subsequent resistance of the malaria parasite to it. However, majority of the respondents (especially the pregnant women) were aware of IPTp with SP and its benefits, including reduction of maternal morbidity and stillbirths, improved weights of babies "*It protects the mother and the baby against malaria infection" *said a pregnant woman in the FGD; "*It makes both the mother and the baby to be healthy" *said another pregnant woman in the FGD. They normally get educated on the IPTp during visits to antenatal clinics and at community durbars and electronic media including radio of which majority, 85% of the women owned.

In spite of the little or no formal education, low socioeconomic status and poor housing units of most (72%) of the pregnant women with over 60% being traders and farmers, the patronage of SP in IPTp was good and higher among multigravid women (58%) as compared to the secundigravid (24%) and primigravid women (18%). The low patronage by the primigravid women as found in the previous studies [9] is discouraging since they are the vulnerable group with high maternal morbidity and low birth weight deliveries [2,13,18,19].

### Study limitations

Helminthic infection, malnutrition and other disease conditions could contribute to anaemia in pregnancy [9] aside malaria, and these were not determined. For the purpose of this study, SP doses administered to the pregnant women were obtained from the ANC cards at the health facilities, and possible non-recording of doses might have led to an underestimation of doses received.

The acceptance/ownership of ITNs as a means of preventing mosquito bites was quite encouraging since over 50% of the pregnant women studied had ITNs although less than 50% admitted usage of the nets. However, the use of ITNs by the pregnant women in combination with the IPTp programme was not assessed since there were chequered responses to the use of the nets. However, these are not expected to significantly influence the observed findings.

## Conclusions

Results of the present study, thus, suggest that effective implementation of the IPTp using SP is an evidence-based measure for control of malaria-related anaemia in pregnancy. Reduced maternal anaemia impacts positively on both maternal and neonatal health. The Ghana Health Service should, therefore, design and implement interventions to increase the proportion of pregnant women (especially primigravid women) who take the recommended three doses of SP during pregnancy.

## Competing interests

The authors declare that they have no competing interests.

## Authors' contributions

EOT, EB, BL designed the study. EOT conducted the research in the field and analyzed the data and wrote the paper. EB and BL advised on the study and reviewed drafts of the manuscript. All authors read and approved the final manuscript.
